# R on the side of caution

**DOI:** 10.1007/s12471-021-01630-2

**Published:** 2021-10-12

**Authors:** L. Baris, E. J. van den Bos

**Affiliations:** Albert Schweitzer Hospital, Dordrecht, The Netherlands

## Answer

The rhythm seen on the electrocardiogram (ECG) is a slow ventricular tachycardia (VT) with a cycle length of 640 ms originating from the left ventricular (LV) lateral wall.

Due to advanced heart failure with severe biventricular dilation and the underlying *LMNA* gene mutation, there is significant intraventricular conduction delay. This causes a remarkable latency in right ventricular (RV) sensing after the onset of ventricular depolarisation in the left lateral wall during this VT.

**Fig. 1:**
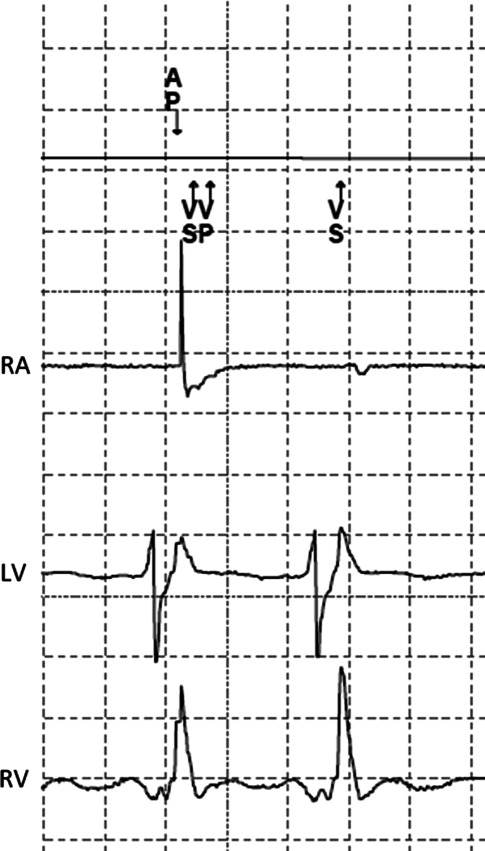
ICD electrocardiogram showing the marker channel above and the right atrial (*RA*), left ventricular (*LV*) and right ventricular (*RV*) channels. Markers indicate a paced atrial event (*AP*), ventricular sensing (*VS*) and ventricular pacing (VP, combined to VSVP ventricular safety pacing) and a ventricular sensed event (*VS*)

With the lower rate set at 80 beats/min (750 ms cycle length), a paced atrioventricular (AV) interval of 200 ms and atrial based timing, an atrial pace spike occurs 550 ms after a ventricular sensed event. Therefore atrial pacing occurs during ventricular depolarisation of the LV wall and an atrial pacing spike is seen in the QRS complex on the surface ECG. Shortly after the atrial pacing spike the ventricular depolarisation reaches the RV lead and is sensed. Due to ventricular sensing within the AV interval, ventricular safety pacing (VSP) is delivered with a short AV delay of 100 ms, as seen in Fig. 1. VSP is a safety mechanism that is activated in case of possible cross-talk by delivering ventricular pacing shortly after atrial pacing if a ventricular sensed event occurs after the ventricular blanking period within the programmed AV interval. Thereby it prevents ventricular asystole. Furthermore, the short AV delay prevents pacing in the vulnerable period of ventricular depolarisation (‘R on T’ phenomenon) in case the sensed event was a premature ventricular beat.

**Fig. 2:**
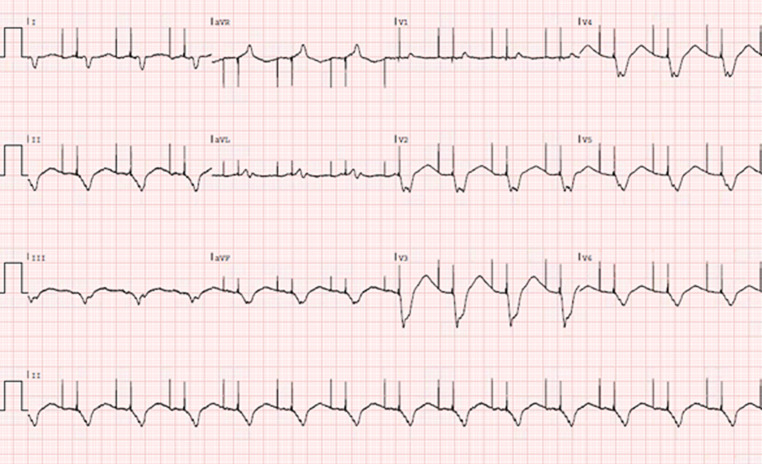
Electrocardiogram showing atrioventricular pacing after electrical cardioversion

After electrical cardioversion, normal AV pacing was re-established (Fig. 2).

